# Editorial: Growth regulation in horticultural plants: new insights in the omics era

**DOI:** 10.3389/fpls.2023.1305520

**Published:** 2023-10-20

**Authors:** Chenxia Cheng, Nan Ma, Yi Zheng, Lin Xi

**Affiliations:** ^1^ College of Horticulture, Qingdao Agricultural University, Qingdao, China; ^2^ Beijing Key Laboratory of Development and Quality Control of Ornamental Crops, Department of Ornamental Horticulture, China Agricultural University, Beijing, China; ^3^ Beijing Advanced Innovation Center for Tree Breeding by Molecular Design, Beijing University of Agriculture, Beijing, China; ^4^ Plant Science and Technology College, Beijing University of Agriculture, Beijing, China; ^5^ Department of Plant Systems Biology, University of Hohenheim, Stuttgart, Germany

**Keywords:** horticultural plants, high-throughput sequencing technology, multi-omics, growth regulation, system biology

In the recent decades, the rapid advancement of next-generation sequencing and "omics" technologies has brought about a revolutionary transformation of the speed and effectiveness of identifying valuable molecules during physiological changes in horticultural plants. A wealth of data on plant genomes, transcriptomes, proteomes, and metabolomes have been generated, enabling researchers to systematically decipher the regulatory mechanisms governing plant growth, development, and stress response. This comprehensive understanding facilitates plant breeding aimed at improving the agronomic traits of interest to breeders and scientists in the community, such as enhancing the yield and quality of horticultural products. Moreover, given the immense diversity of horticultural species and their diverse histories of cultivation, multi-omics studies can offer novel insights into the evolution of various traits and their domestication history. The current Research Topic, titled “Growth Regulation in Horticultural Plants: New Insights in the Omics Era” aims to leverage the potential of high-throughput omics approaches to uncover the molecular mechanisms governing growth and development in horticultural plants. Within this Research Topic, 13 studies authored by 111 researchers contribute valuable insights into multi-omics investigations concerning genes, gene networks or metabolites that regulate growth and development in horticultural plants. Among these studies, nine focus on fruit crops, one centers on vegetable and three delve into ornamental crops.

## Research on fruit crops

Apples are highly popular and widely consumed fruits known for their nutritious, sweet and sour flavors. Consequently, traits associated with apples’ commercial values are of great interest to breeders. These traits encompass aspects such as fruit quality, resistance to pathogens, propagation efficiency, regulation of flowering, and more. (Luo et al., 2020; Liao et al., 2021; Milyaev et al., 2021; Fan et al., 2022). Among these aspects, the appearance of fruit plays a crucial role in the evaluation of apples. In this Research Topic, Wang et al. conducted transcriptome analysis, chemical staining, and LC- and GC-MS analyses to uncover the factors responsible for the formation of russet apple fruit. Their findings highlighted that the elevated levels of lignin and suberin were key contribution to russet formation. Additionally, the study identified several metabolites and genes associated with this process. Virus infections have a significant impact on sustainable development of apple production as well as the fruit’s quality (Hadidi et al., 2011). Apple scar skin viroid (ASSVd) leads to a decrease in apple fruit size, causes scarring and staining of the apple peel. These effects can subsequently diminish the marketability of apple fruits and have adverse implications for the apple industry. Li et al. shed light on the underlying mechanisms of ASSVd infections and emphasized the importance of virus-free cultivation for sustainable crop production, as demonstrated through leaf metabolomics. For many fruit varieties, including apple rootstocks, the lack of adventitious root formation presents a challenge for their asexual propagation. Tian et al. pointed out that *MdWRKY87* promoted adventitious rooting through regulating root-related gene involved in auxin signaling pathway by the comparative transcriptome profiling. Breeders have been intrigued by the regulatory mechanisms of floral initiation of cultivated apple and other perennial woody plants, with gibberellins (GAs) identified as key regulators. The function of GA2ox, an enzyme responsible for modulating the levels of active GAs, plays a crucial role in this process. Zhang et al. conducted a comprehensive investigation to elucidate the biological and evolutionary perspectives of GA2ox in apple, focusing specifically on its role in floral induction. The study revealed that the *MdGA2ox2A/2B* genes potentially participate in the repression of flowering.

Grape is among the top fruit crops cultivated worldwide. As a prominent fruit used in wine production, the diversity of compounds from various Vitis species has attracted cultivators and scientists attention (Lin et al., 2019). A noteworthy study by Cheng et al. focused on *Vitis adenoclada*, a unique wild species endemic to China. They successfully assembled a high-quality, chromosome-level genome for *V. adenoclada* with a 498.27 Mb in size and 28,660 protein-coding genes. Through comprehensive metabolome and transcriptome analysis, the research uncovered the higher expressions of COMT and STSs genes linked to increased phenolic compound content, subsequently impacting the sensory attributes of grapes and wine. Furthermore, when serving as table or dried fruit, grape quality primarily hinges on sugar composition and content. In China, the Eurasian grape resources from Xinjiang are very rich in diversity. Zhong et al. utilized GC-MS to determine the sugar components and content in 18 grape varieties, offering effective ways to enhance sugar content through breeding. Grapes are also among the oldest fruit crops cultivated worldwide. Consequently, a range of genomics knowledge is essential for either comprehending the domestication process or developing effective strategies for crop improvement. (Badouin et al., 2020; Navarro-Paya et al., 2021). Xiang et al. conducted a systematic genome-wide and expression analysis of sixty RNA directed DNA methylation (RdDM) pathway genes in grapes and predicted their involvement in multiple biological processes. This study introduced new candidate gene resources for further functional characterization and molecular breeding of grapes.

Citrus, a globally important evergreen fruit tree species, is another major consumable fruit provider, offering fruits as well as diverse by-products. However, it is susceptible to waterlogging, which leads to reduced yields. He et al. employed RNA-seq to identify 17 differentially expressed transcription factors (TFs) that play a pivotal role in enhancing citrus’ tolerance to waterlogging.

Blueberries, known for their deliciousness and health benefits, are renowned for their high anthocyanin content. Light plays a significant role in plant growth and development. Zhang et al. conducted an integrated transcriptome and metabolome analysis and found that blue light stimulated the expression of LDOX, UFGT, and OMT genes, leading to the accumulation of cyanidin, pelargonidin, and malvidin anthocyanidins. On the other hand, the combination of red and blue light resulted in the up-regulation of DFR and OMT genes, promoting the accumulation of various downstream metabolites, including delphinidin, petunidin, and peonidin derivatives.

## Research on vegetable crops

Chinese cabbage (*Brassica rapa ssp. pekinensis*) holds significant economic importance as a vegetable in Asia and is now widely available in markets around the world (Sun et al., 2022). Leaf size is a critical trait that directly affects Chinese cabbage yield. In a study conducted by Wang et al., transcriptome analysis revealed the pivotal roles played by cyclins (CYCB and CYCD) and transcription factors (MYB47 and MYB88) in regulating cell proliferation, thereby influencing leaf size variation. The findings of this study provide valuable insights for future endeavors aimed at improving Chinese cabbage yield through molecular breeding approaches.

## Research on ornamentals

Chrysanthemum, renowned globally for its wide range of intricate flower types, holds immense value in molecular breeding for enhancing floral characteristics. It ranks second in the cut flower trade, following the rose (Su et al., 2019). Li et al. embarked on unraveling the molecular mechanisms underlying chrysanthemum flower development. They identified a total of 44 MIKCc-type MADS box genes throughout the whole genome-wide level in *Chrysanthemum lavandulifolium*. Through their investigation, they observed the expression of C-class genes in the corolla of disc florets, offering potential insights into the morphological distinctions between disc and ray florets. These findings contribute to the establishment of a tetrameric model elucidating floral organ development in *C. lavandulifolium*.

Another globally popular cut flower is the lily. However, it is sensitive to high temperatures (Ding et al., 2021). Among its cultivars, Lanzhou lily (*Lilium davidii* var. unicolor), is a well-known edible crop cultivated in China. Cao et al. conducted an RNA-seq analysis to gain insights into the thermotolerance mechanisms of Lanzhou lily induced by trichokonins. Their findings suggest that LzHsfA2a-1 plays a crucial role in the acquisition of thermotolerance when exposed to trichokonins. This is attributed to its sustained response to heat stress and enhanced response to trichokonins treatment during prolonged heat stress.

*Catalpa fargesii* is a native tree species in China, valued for both its timber and ornamental qualities (Yu et al., 2022). Maiyuanjinqiu, a new variety of *C. fargesii*, exhibits significantly lower total chlorophyll content and reduced photosynthesis compared to the original species. Zhang et al. discovered that the m6A methylation level of total RNA was higher in yellow leaves than in green leaves. By silencing the methyltransferase gene *CfALKBH5*, the researchers induced a chlorotic phenotype and increased m6A methylation levels. These findings underscore the significance of m6A modification as an epigenetic mark associated with leaf color in *C. fargesii* and provide valuable insights for manipulating the epitranscriptome to enhance natural leaf color variation in forest breeding.

In summary, the studies mentioned above aim to achieve desirable traits, laying the groundwork and showcasing significant recent progress in identifying novel targets for genetic modification in horticultural plants. Multi-omics studies offer fresh perspectives into understanding the genetic mechanisms underlying crucial plant characteristics. The utilization of omics strategies in plant genetics and breeding research presents both promising opportunities and challenges.

## Author contributions

CC: Conceptualization, Writing – original draft, Writing – review & editing. NM: Writing – review & editing. YZ: Writing – review & editing. LX: Conceptualization, Writing – original draft, Writing – review & editing.
